# Method for measurement of collagen monomer orientation in fluorescence microscopy

**DOI:** 10.1117/1.JBO.26.7.076501

**Published:** 2021-07-08

**Authors:** Rodrigo P. Alzola, Seyed Mohammad Siadat, Anuj Gajjar, Rickard Stureborg, Jeffrey W. Ruberti, Jose Delpiano, Charles A. DiMarzio

**Affiliations:** a Northeastern University, Department of Electrical and Computer Engineering, Boston, Massachusetts, United States; b Universidad de los Andes, Optical Communications Lab, School of Engineering and Applied Sciences, Santiago, Chile; c Northeastern University, Department of Bioengineering, Boston, Massachusetts, United States; d Northeastern University, Department of Mechanical and Industrial Engineering, Boston, Massachusetts, United States

**Keywords:** collagen, monomer orientation, fluorescence microscopy, Gabor filter, convolutional neural network

## Abstract

**Significance:** Collagen is the most abundant protein in vertebrates and is found in tissues that regularly experience tension, compression, and shear forces. However, the underlying mechanism of collagen fibril formation and remodeling is poorly understood.

**Aim:** We explore how a collagen monomer is visualized using fluorescence microscopy and how its spatial orientation is determined. Defining the orientation of collagen monomers is not a trivial problem, as the monomer has a weak contrast and is relatively small. It is possible to attach fluorescence tags for contrast, but the size is still a problem for detecting orientation using fluorescence microscopy.

**Approach:** We present two methods for detecting a monomer and classifying its orientation. A modified Gabor filter set and an automatic classifier trained by convolutional neural network based on a synthetic dataset were used.

**Results:** By evaluating the performance of these two approaches with synthetic and experimental data, our results show that it is possible to determine the location and orientation with an error of ∼37  deg of a single monomer with fluorescence microscopy.

**Conclusions:** These findings can contribute to our understanding of collagen monomers interaction with collagen fibrils surface during fibril formation and remodeling.

## 1 Introduction

Collagen is the predominant fibrillar component and the most abundant protein in vertebrate animals.^1^ Its molecular unit, ∼300  nm long with a diameter of 1.5 nm,^2^ is composed of three α-chains assembled into a triple-helix.^3^ In an adult tissue, collagen molecules are assembled into 20 nm to a few hundred nanometers diameter fibrils^4^ in a quarter-staggered arrangement.^5^ These fibrils are the fundamental structural unit in a variety of load-bearing tissues, such as bone, tendon, ligament, blood vessel, skin, and cornea.^6^ Although fibril assembly and remodeling have been widely investigated, their underlying molecular mechanisms are yet poorly understood.^7^ Specifically, the detailed mechanism by which the collagen molecules are added to the collagen fibrils is not known. We are thus interested in visualization of free-collagen monomers interacting with the fibril surface.

Though collagen fibrils themselves are readily imaged via light^8^ and electron microscopy,^9^ less imaging has been done on monomers and their dynamic interaction with the fibril surface. To obtain an image of a collagen monomer, electron microscopy is often used.^10^ The limitation of this detection technique is that as a result of the sample preparation processes the samples are destroyed. Thus, electron microscopy allows us to observe monomer orientation only statically on a fixed sample. Detecting location and orientation dynamically is important in identifying the mechanisms that promote or suppress the molecular assembly of collagen.

Fluorescent labels can be attached to molecules and excited with short wavelength light so that it is possible to locate them by imaging the emitted light at a longer wavelength. The chemical structure of the monomer is such that there are multiple sites where a fluorophore can attach. We have used this opportunity and previously showed that, with increasing fluorophore density, we can control the average number of labels per each monomer.^11^^,^^12^ Labeled monomers functionality tests showed that monomers labeled with more than two tags are significantly disrupted and do not participate in the collagen fibrillogenesis process. However, adding two or less labels mostly preserved the labeled monomers functionality.^11^^,^^12^ Furthermore, we introduced single molecule, multilabel fluorescence orientation microscopy to detect collagen monomers orientation with multiple labels.

When the fluorophores are located at two points along the collagen monomer, the separation is smaller than the resolution. Therefore, the epi-fluorescence image is the incoherent superposition of two point-spread functions (PSFs) of the microscope at the emission wavelength with each one centered on one of the two fluorophores. Since the separation is smaller than the resolution, the image can be approximated into an ellipse where the major axis represents the collagen molecule orientation. To identify orientation, at least two fluorescent labels must be attached to each sample as one label is only sufficient to determine location. There are 38 positively charged and hydrophilic lysines and one N-terminal α-amine as potential binding site on each α-chain for the label. In our tagging approach, monomeric collagen was labeled at pH 7.5 with amine-reactive dyes to increase the likelihood of attaching a fluorophore at the N-terminal of the monomer and consequently maximize the separation between the N-terminal bound fluorophore and a secondary fluorophore closest to the C-terminal. Since monomers labeled with more than two tags significantly disrupt monomer functionality,^11^^,^^12^ we limited our investigation to monomers labeled with only one or two fluorophores. In this study, unlabeled monomers were not observed (due to lack of fluorescent signal) and singly labeled monomers were located but orientation was not determined. We considered the doubly tagged monomers and developed the first nondestructive technique to detect their orientation under epifluorescence microscopy.

First, we developed a simulation of the optical system to generate synthetic visualizations of collagen monomers. We then used this simulation to generate a dataset of artificial images, with synthetic monomers with degrees between [−90  deg, 90 deg]. The spacing of the fluorophores was varied randomly between 150 and 300 nm to simulate a variety of binding sites for the second fluorophore. An average background level was computed based on scattering of light that subsequently leaks through the filters. This level could be increased to account for out-of-focus fluorophores and autofluorescence for unlabeled monomers. Along with the signal photons, this mean background was subjected to the Poisson distribution. The mean background was subtracted for processing, but the noise associated with the Poisson distribution remained. In one algorithm, these images were used to train a classification model with a convolutional neural network (CNN). Machine learning approaches have been used to classify collagen in space.^13^ However, previous studies generate models to give a diagnosis as an output for healthy or unhealthy collagen. This requires a higher composition level of collagens as fibrils and higher observation dimensions around 10  μm. This model was used to distinguish the difference between single- and double-tagged monomers and in the latter case its orientation. In the second algorithm, Gabor filters were used to compare performance and accuracy. Both algorithms were tested and compared using synthetic data. Finally, we tested the performance of these models in detection of position and orientation of labeled monomers, which were absorbed on glass.

## 2 Methods

### 2.1 Collagen Labeling

Type I collagen solution (5026-50ML, TeloCol, Advanced BioMatrix) was used for the study and labeled with AF488 (Alexa Fluor 488 TFP ester, A37570, Invitrogen) as described previously.^11^^,^^14^ Briefly, collagen monomers were diluted in 10 mM HCl to 1  mg/mL and then mixed 1:1 with 0.2 M sodium carbonate–bicarbonate buffer (24095, Polysciences) containing 1 M sodium chloride (S671-3, Fisher Scientific). AF488 was dissolved in dimethyl sulfoxide (D12345, Life Technology) to a concentration of 0.5  mg/mL and added to collagen solution in excess amount to achieve at least two fluorophores on average on each collagen monomer. The reaction mixtures were stirred for 3 h at room temperature. Labeled monomers were dialyzed in dialysis cassettes (Slide-A-Lyzer, 3.5K MWCO, 3 mL) for 3 days in 10 mM HCl. Collagen concentration was measured using DC Protein Assay (Bio-Rad). AF488 concentration was measured using Beer–Lambert law. Degree of labeling was measured as moles of fluorophore per mole of collagen.

### 2.2 Optical System

**Fig. 1:**
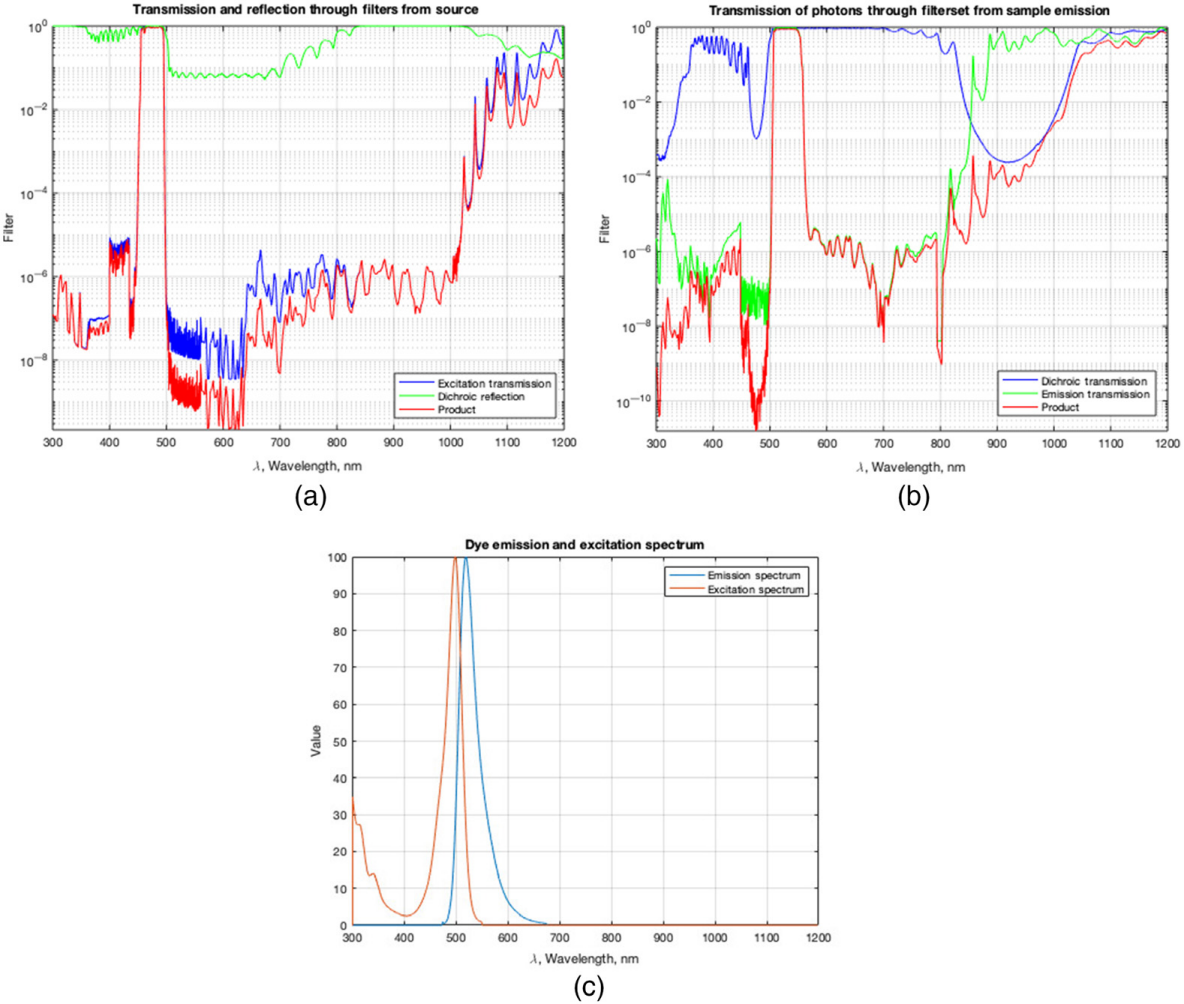
Transmission and reflection spectra of the Nikon 96311 B-2E/C filter cube that is obtained from the manufacturer. (a) Light from the source is transmitted through an emission filter and reflected by the dichroic mirror. (b) Light emitted by the sample is transmitted through an excitation filter and dichroic mirror. (c) Emission through the excitation spectrum that is obtained from the manufacturer of the AF488 fluorescent dye.

The sample was illuminated with a blue LED light source [ThorLabs M470-L3-C5, 470 nm, 650 mW (minimum) LED]. The filter set used was a Nikon Fluorescence Cube (96311 B-2E/C). The excitation filter and dichroic reflection on this cube provide a pass-band excitation filters of blue light (465 to 495 nm wavelengths) as shown in Fig. 1(a) (data provided by the manufacturer). Wavelengths that pass through the filter and are reflected by the dichroic mirror are focused onto the sample with a 60× objective lens (Nikon’s CFI Apochromat TIRF Series, numerical aperture: 1.45) and fluorescent emission is detected through the same lens. Fluorescence from the sample is transmitted through the dichroic and emission filters with a pass-band for green light (515 to 555 nm wavelengths). Most of the emission spectrum is passed through this filter as shown in Fig. 1(c). From Fig. 1(b), we note that the transmission of any scattered or reflected excitation light at 470 nm is reduced by nearly 10 orders of magnitude. The microscope was a Nikon inverted microscope (ECLIPSE TE2000-E) that used two cameras: CoolSNAP EZ CCD Camera (1392×1040 imaging array with 6.45-μm square pixels). The images obtained by the cameras were taken using the Nikon NIS-Elements software.

### 2.3 Image Simulation

The CoolSNAP EZ CCD camera has a resolution of 6.45  μm per pixel, with a 60× objective the resolution results in 110 nm per pixel. A collagen monomer at 300 nm long only subtends a few pixels at best. However, due to diffraction, a fluorophore, which can be considered a point object, is imaged as the PSF, of diameter:

$$\mathrm{PSF}=1.22\frac{\lambda }{\mathrm{NA}},$$

in which NA=1.45 is the numerical aperture of the objective and λ is the wavelength, centered at 530 nm. PSF is measured as the diameter of the first dark ring. Thus, for a single fluorescent tag we expect a spot size of about 450 nm. We developed a simulation of collagen images. The simulation and all the image processing were performed with MATLAB, 2019.

The first step of the simulation was to determine the number of electrons the sensor produces from signal, background, scattered light, and noise. The radiance spectrum of the blue light source (470 nm) was multiplied by the transmission through the excitation filter and reflection through the dichroic filter [Fig. 1(a)]. Then, the above spectrum was converted to photon spectral irradiance rate using the photon energy and the solid angle of the objective lens, in which the solid angle Ω was calculated by

$$\Omega =2\pi (1-\sqrt{1-\frac{{\mathrm{NA}}^{2}}{n^{2}}}),$$

in which n=1.56 is the refractive index of the glass medium that holds the sample. Thus, the photon spectral irradiance was calculated using the equation:

$$\text{Photon spectral irradiance}=\frac{hc}{\lambda }\cdot \Omega \cdot \text{spectral radian},$$

in which h is the Planck constant and c is the speed of light. This photon spectral irradiance is the number of photons per unit time and area illuminated onto the sample. This quantity was multiplied by the absorption cross-section of the fluorescent tag $1.212\times 10^{-20}\;m^{-2}$, (provided by the manufacturer) of the AF488 dye to determine the rate of the number of photons absorbed by the sample. Using the quantum yield and lifetime of the dye, the rate of photons emitted by the sample entering the camera was determined as

$$\text{Photon emission rate }(\mathrm{PER})=\frac{\Omega }{4\pi }(\frac{\phi }{\frac{1}{\gamma }-t}),$$

in which ϕ is the quantum yield, γ is the photon absorption rate, and t is the lifetime. The emission rate was then multiplied by Ω/4π, the fraction of light collected in the solid angle Ω of the objective and the transmission spectra of the emission filter and dichroic mirror in Fig. 1(b). The number of electrons excited in the sensor from the emission of the sample:

$$N_{e}=t\int \mathrm{PER}*\mathrm{QE}d\lambda ,$$

where the quantum efficiency (QE) spectrum of the camera system is provided by the manufacturer and the integral is over wavelength.

We then simulated the number of electrons in the sensor from the scattered light and background noise. In our simulation, we estimated a background level of light caused by excitation light that passed through the dichroic and emission filters to reach the detector. The background was considered as a number of photons per pixel and could include other sources such as out-of-focus fluorophores and autofluorescence. To model leakage of the excited light, a similar process was used except that instead of calculating fluorescence from absorption and quantum yield, we multiplied the photon irradiance by the scattering section of the AF488 dye, estimated as $1.1\;e^{-13}\;m^{-2}$. The rest of the process is the same, but now the wavelength is still that of the source, so the transmission through the emission filters is lower. Background light can also be added. The noise is determined by the exposure time multiplied by the readout RMS and dark current values provided by the manufacturer plus electrons from scattering. It is important to note that these signal and noise values do not account for the autofluorescence of the sample, scattered light that leaks in the camera, and residues of fluorescent material.

**Fig. 2:**
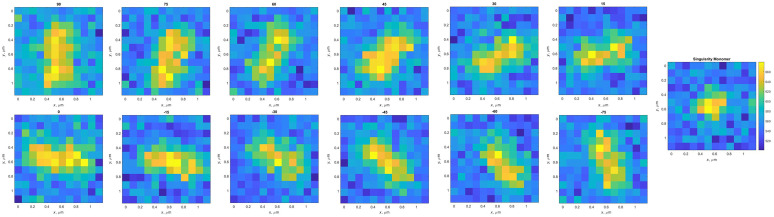
Simulation for collagen monomers: first generation of fluorescence point sources and defining a PSF determined by our optical setup. Then, the point sources are seen by the diffraction-limited system after the aperture. The resulting image representing the accumulation of photons in the camera is corrupted with Poisson noise. Result in a dataset labeled by different orientations including singularity monomers. Colormap indicates electron counts.

To generate a synthetic image, a random pixel position was chosen to emulate one of the fluorophores with the value of the signal in electron counts, in a resolution 10 times smaller than the original dimensions. To simulate a double-labeled monomer, a second fluorophore was located at a random distance from the other, at a maximum of 300 nm down to 150 nm, with a random orientation between [90 deg, −90  deg] in intervals of 15 deg. We chose to limit the minimum separation of fluorophores to 150 nm. The pixel size dictated by our camera and objective is 110 nm, so we do not expect reliable measurements of double-labeled monomers below this limit. Nevertheless, we will image about half of the double-labeled monomers, providing a useful sample of monomer orientation. With random locations for the second fluorophore, about half of the double-labeled monomers would be included within these dimensions, enabling a statistical analysis of orientation in a sample of collagen monomers. The image was convolved with the PSF that represents the optical transfer function. The image was then resized to its correct dimension. Finally, a Poisson distribution was applied to the total number of electrons, resulting in the final image (Fig. 2).

### 2.4 Bank of Gabor Filters for Determination of Orientation in Monomers

**Fig. 3:**
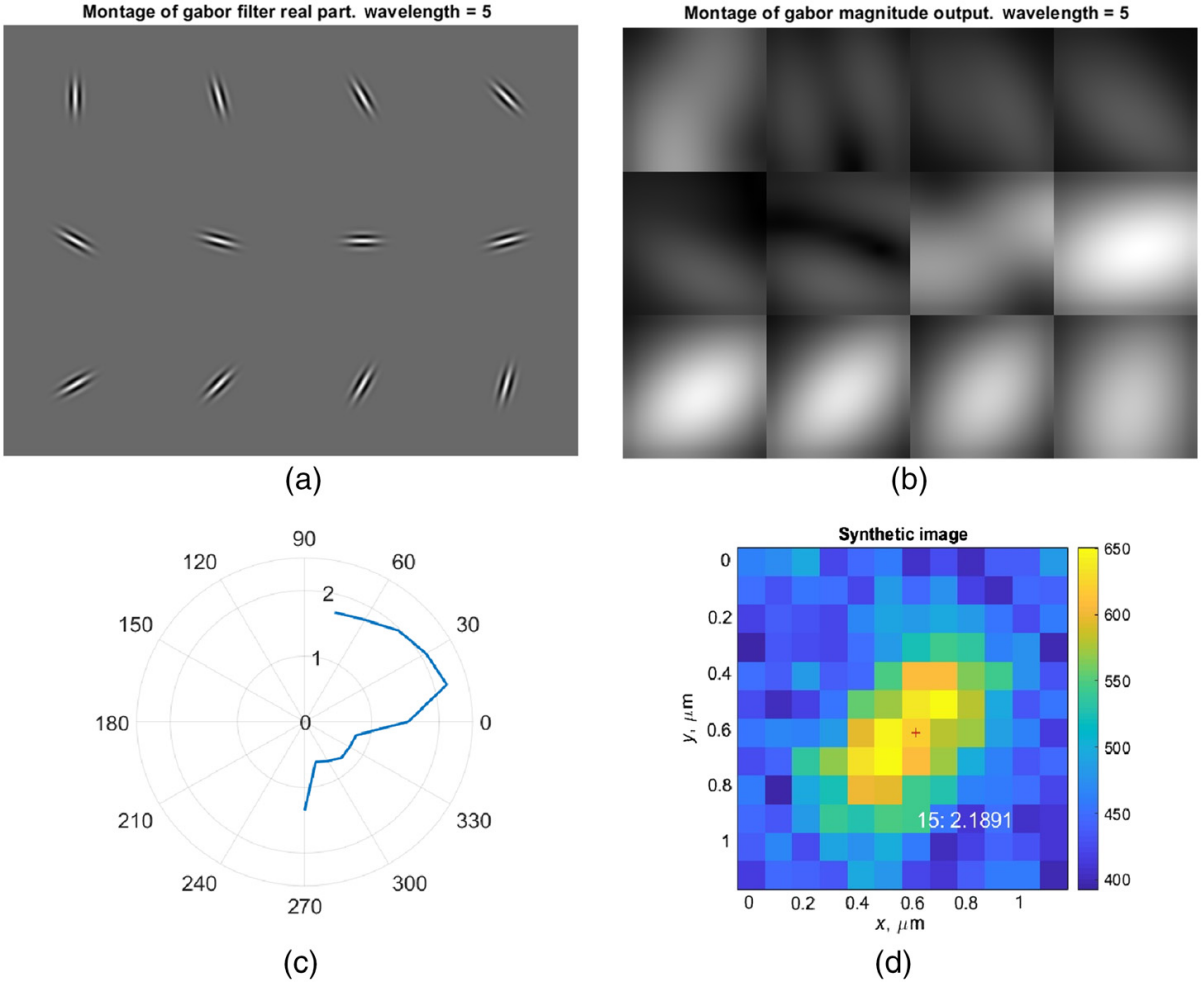
Steps for detection of orientation with a bank of Gabor filters. (a) Montage of real part of a bank of Gabor filters with wavelength of 5. (b) Magnitude of Gabor output for the results of applying all the sample filters to a monomer image. We will also call this the Gabor feature, which is a function of spatial position in both axes and the orientation of the specific filter in the bank. (c) Polar plot of maximum Gabor feature for each orientation, finding the degree for the orientation. (d) Input monomer image and superimposed position and detected orientation. Detected orientation is 15 deg, followed by 2.1891 being the maximum value of the corresponding Gabor feature for the overlap of the image with the filter. Colormap indicates electron counts.

Gabor filters are linear filters that are well-suited for quantification of given spatial frequency components at specific locations and orientations in a two-dimensional signal.^15^^,^^16^ Figure 3 shows the steps of the algorithm we devised to estimate monomer orientation with Gabor filters. A simulated input image, with one double-tagged monomer is enhanced by the nonlinear “block-matching and 3D filtering” denoising method.^17^ Samples of the filters in the bank are chosen [Fig. 3(a)] and show all feature maps resulting from convolution of the enhanced image and all sample filters [Fig. 3(b)]. Gabor feature is the result of the product of the image with the filter, being the value different for each of the filters in the bank. The maximum value of the Gabor feature is plotted for each orientation [Fig. 3(c)]; the maximum degree is shown in perpendicular orientation. The final orientation estimation is superimposed upon the input image [Fig. 3(d)]. A Gabor filter cannot detect a monomer that is singularly labeled, as the pattern is expected to be circularly symmetric, and thus all the filters will have similar results except for variations caused by the Poisson distribution and noise. For this reason, an initial decision must be made based on the area to discern if the monomer is one or two labeled. To determine pixels that are part of the monomer area, a value for each pixel of 175 counts is selected as threshold. If the value is over this threshold it can be considered as part of the signal and not noise or background, being able to calculate the area of a monomer. For the modify Gabor filter, if the area has less than seven pixels above threshold it will be classified as a single monomer.

### 2.5 CNN for Detection of Monomer Orientation

**Fig. 4:**
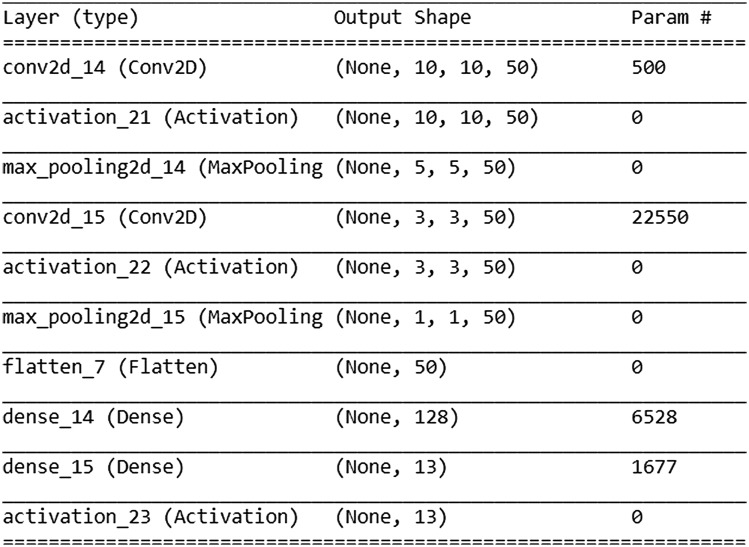
Architecture of CNN having as components two convolutional layers.

In another algorithm to measure the angle of orientation of the monomers, a machine learning approach was taken. A CNN was trained and tested on 26,000 samples of simulated data, divided into 70% for training and 30% for testing. A validation dataset conforms of 6500 samples is used to measure the results in Sec. 3.1.2. The task was trained as a classification problem, with monomers labeled as being within one of 12 classes, each with an angular range of 15 deg. There is also a 13th class that denoted single-tagged monomers. These are monomers that only contain one fluorescent tag and therefore an orientation cannot be determined. We used two convolutional layers with a categorical cross-entropy loss function for the classification task with a calculation of 52,909 parameters. The model was trained in three epochs and the number of samples was 32 for the batch size. Architecture of CNN is described in Fig. 4. Training was made in Python3.6 and Keras2.3.1 in Intel Core i7-8750H CPU @ 2.20 GHz 2.21 GHz machine. Total time in training was 6.87 s. Our use of CNN is distinct from traditional tasks this type of architecture is used to solve. Unexpected orientation of objects can typically fool object detection models, whereas our architecture is trained to measure this very orientation.

## 3 Results

### 3.1 Detection of Monomer Orientation in Simulated Data

#### 3.1.1 Gabor results

**Fig. 5:**
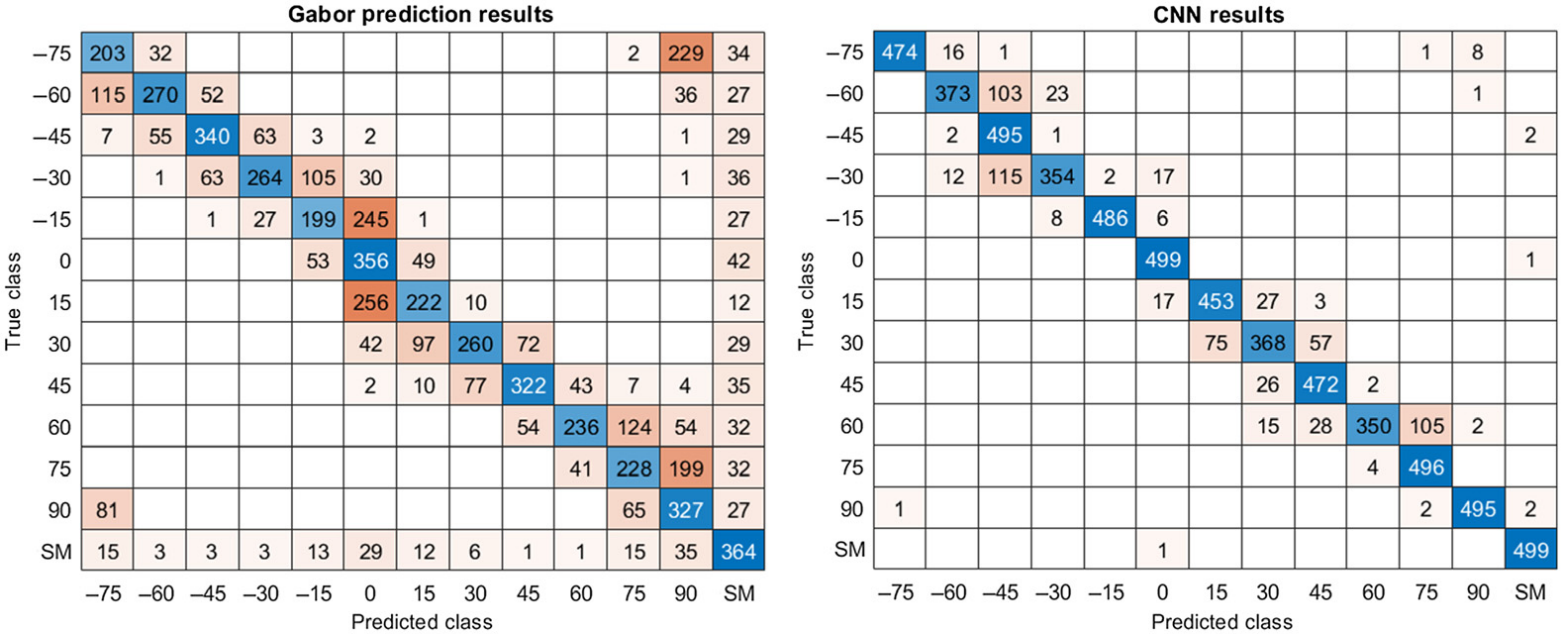
Classification results of synthetic data. Show accuracy and misclassification of the two approaches. (a) Confusion matrix of the Gabor filter set. (b) Confusion matrix of the CNN.

The Gabor filter set has a performance of 55.03% accuracy and when it failed, the misclassification was usually next to the closest orientation leading to an error of 15 deg. If a monomer has a single fluorophore, the filter will try to detect an orientation anyway. The circular symmetry will be broken by pixel-to-pixel variations caused by the Poisson statistics or noise and one of the filters will incorrectly have the best match. To correct this deficiency, an initial decision based on area was made to distinguish between a double and single labeled monomer. For validations 6500 samples were used. The results of classification are presented in Fig. 5(a). Most misclassifications are close to the diagonal, so we can say that Gabor filter has an error of ∼15 deg.

#### 3.1.2 CNN results

The machine learning approach using the CNN produced 89.45% accuracy. This indicates that CNN rarely misclassifies, but it raises concern that the algorithm may be overfitted. When working with the simulated data, high accuracy of a complex CNN may be a sign of overfitting. Here, we understand that a CNN is complex if it has a large number of parameters with respect to the complexity of the space of plausible monomer images. To lower that risk of overfitting, we trained a relatively simple neural network. Even current CNN research for computer vision focused on performance yields models that have a number of parameters in the order of millions.^18^ We did not use real data for training, the most conclusive experiment is to face CNNs to real data. As the trained CNN is still classifying orientation in images of real monomers, we can be sensibly sure that overfitting is not important in our results. Furthermore, the model accurately classified monomer orientation using the same 6500 validation samples as the Gabor filters. The results of classification and misclassification are presented in Fig. 5(b). The success of our model could likely be attributed to the low dimensionality of the layer. That is, the kernels learned are quite simple despite having to do with rotation. However, this learning approach is still more accurate than other Gabor filters in simulated data.^19^

### 3.2 Experiments with Fluorescent Collagen Monomers

**Fig. 6:**
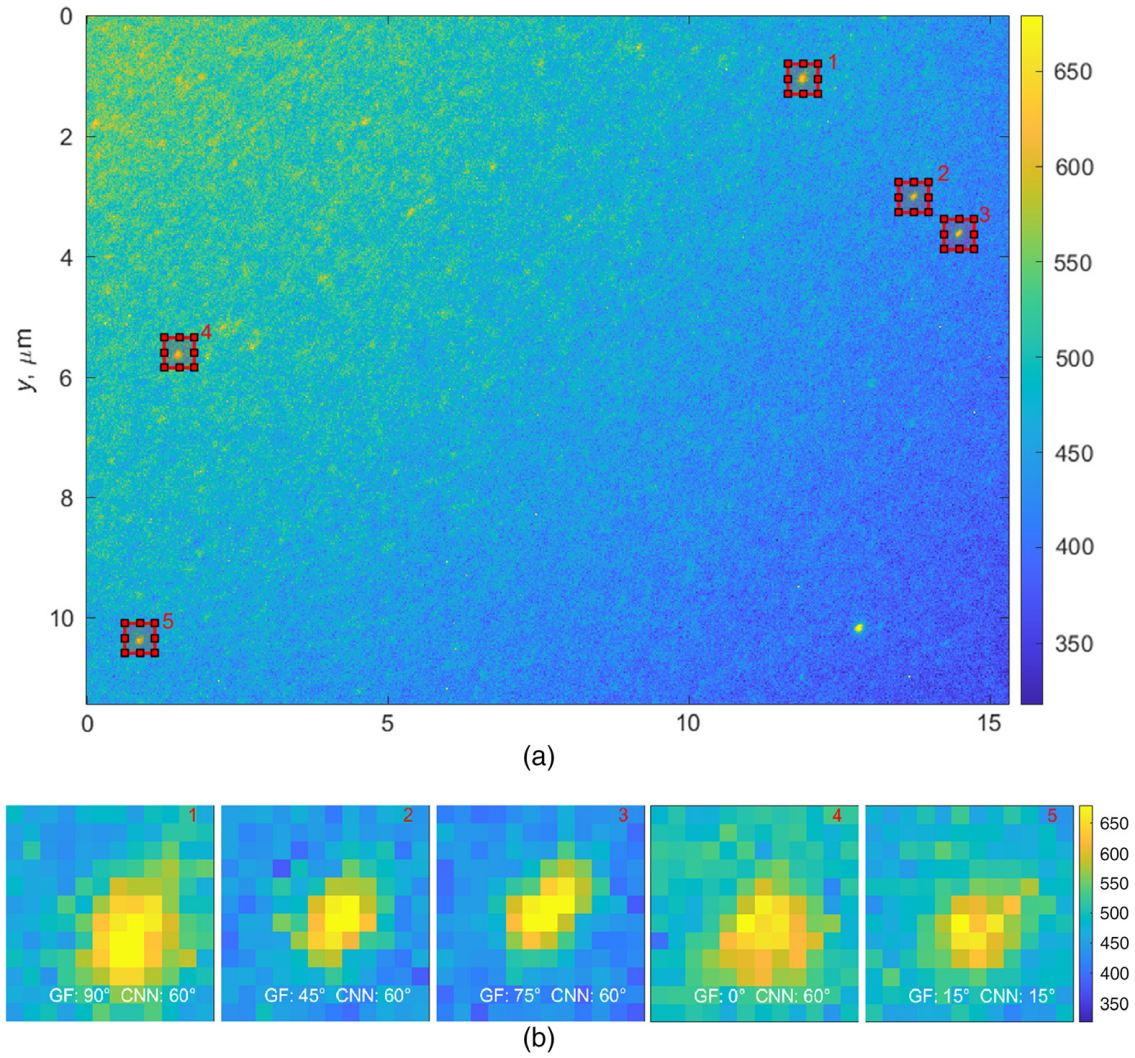
(a) An experimental image using fluorescence microscopy. (b) Five numbered regions of interest show fluorescence detection results for isolated collagen monomers. Region numbers correspond to the numbered locations in (a). Examples show the output of the classification of orientation of Gabor filter and CNN model in degrees.

Next, we applied the two algorithms to data collected from images of labeled monomers, which were absorbed on glass in the laboratory. The samples were from a 314-μg/mL collagen solution with an average labeling of 2.5 tags per collagen monomer. Images were taken with an exposure time of 5 s. For single molecule visualization, samples were diluted 100 to 1000 times to prevent light from saturating the camera and to reduce the fluorescence and scattering from monomers that were out of focus. Monomers in the experimental image were clearly visible so the images were tested to determine orientation (Fig. 6).

### 3.3 Detection of Monomer Orientation in Experimental Data

#### 3.3.1 Detection in isolated monomers

**Fig. 7:**
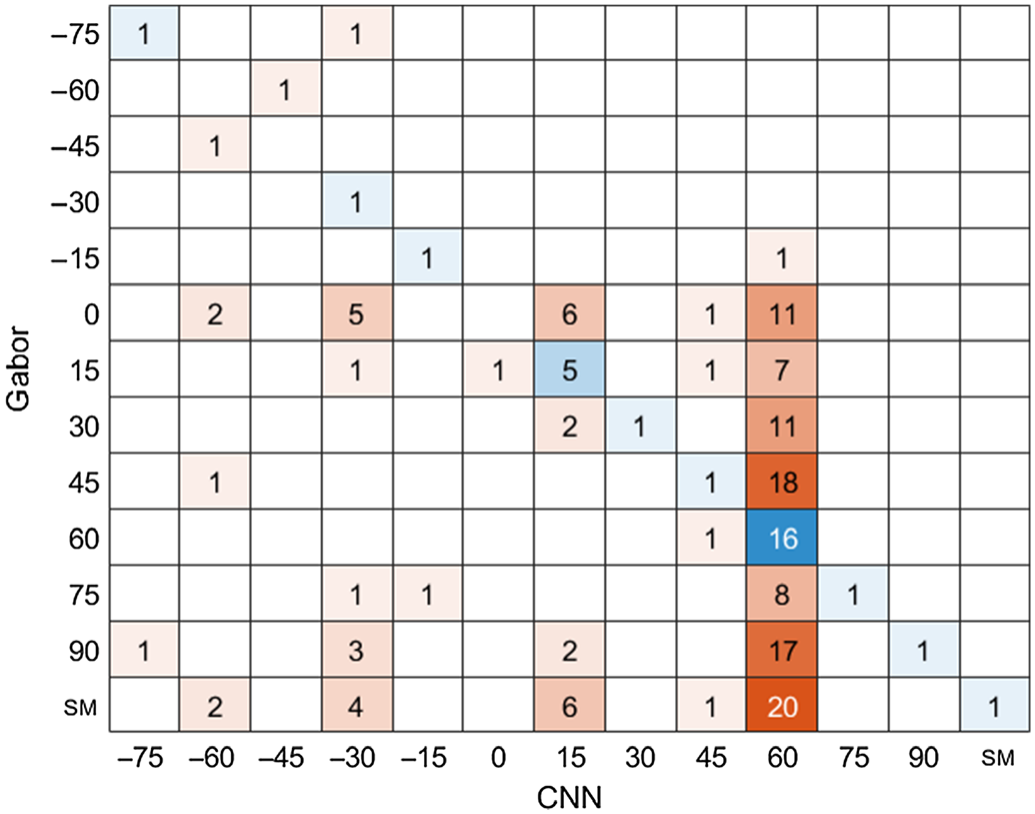
Confusion matrix for the two models. The results for the detection of 154 isolated monomers for Gabor filter and the CNN predictions.

Assuming that all fluorophores were attached to collagen, collagen monomers were detected by the level of signal. In any given image, it was possible to detect several isolated monomers. As shown in Fig. 5, the Gabor filter is more likely to misclassify than the CNN model. One problem is that it is not possible to distinguish between single and double tagged monomers using just the Gabor filter so that decision was based on the area of the image. The most significant discrepancy between the two models can be observed in Fig. 7. When the algorithms differ, there is uncertainty as to which is more correct because, unlike the simulations, the experimental data lack ground truth. Epifluorescence measurement followed by sample preparation and electron microscope measurements would be a challenging task, probably requiring a special instrument with both imaging modalities.

#### 3.3.2 Sequential detection

**Fig. 8:**
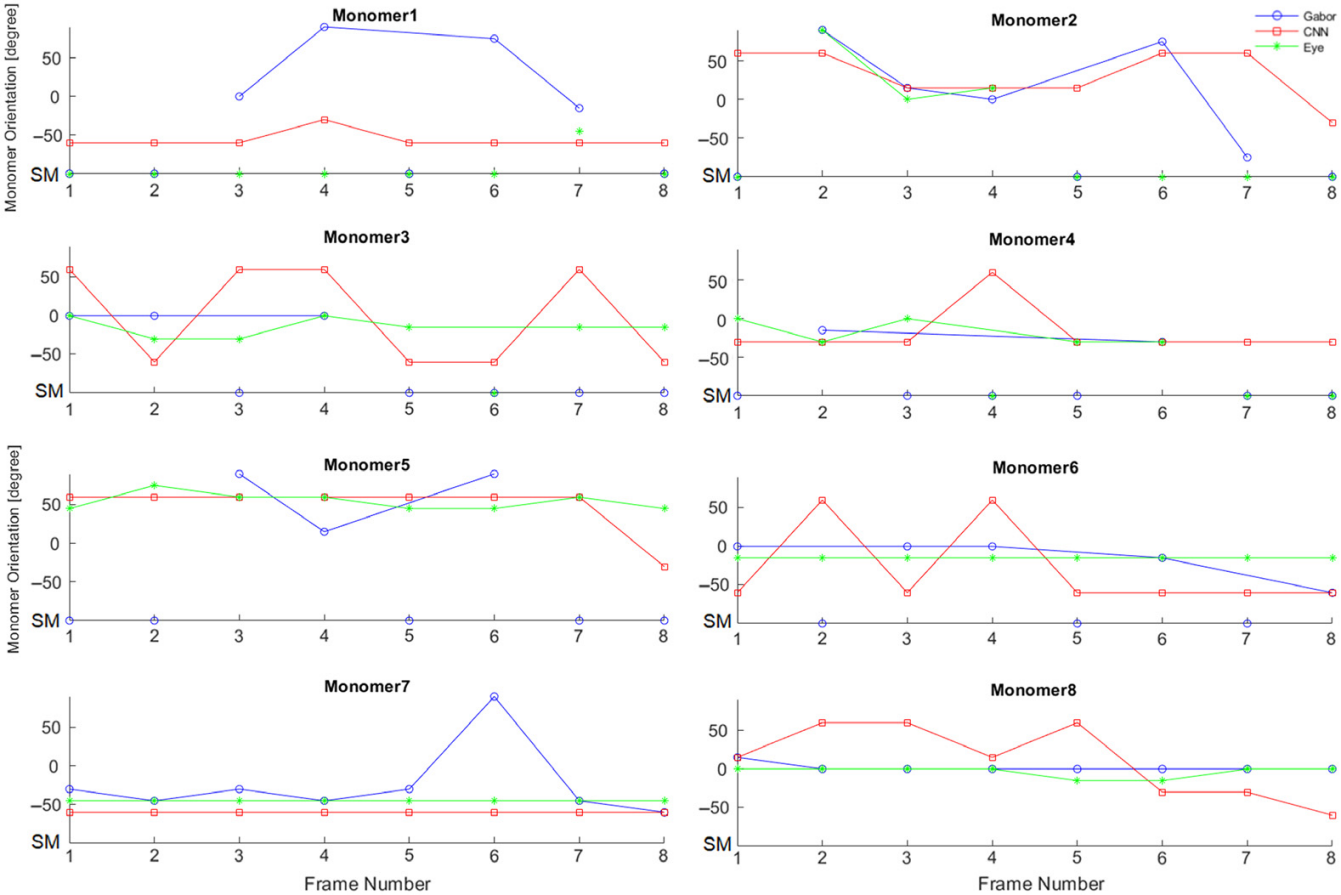
Table of comparison for every timestamp for monomers in the same sample. Images were taken every 10 s. Orientation is classified between [−90 deg, 90 deg] for double labeled monomers and is shown as a continued function. Classification of singularity labeled monomers (SM) is shown as separated points, for the reason of no directionality. Results from the Gabor filter, the CNN model, and the eye observations are shown in blue, red, and green colors, respectively.

In biomedical applications, collagen monomers are usually studied in physiological conditions and in association with collagen fibrils. To address the dynamic interaction of collagen monomers with fibril surfaces, different monomers were analyzed in successive images collected at different times to test the reproducibility of the algorithms under different realizations of Poisson electron statistics and noise. Eight pictures were captured separated in time by 10 s. There is a slight variation from one capture to the next, as the illumination and the noise vary. The results of the algorithms for every isolated monomer through the eight times were compared. Also, a visual classification for each monomer was made, with the purpose of comparing the similarity of the models to human eye observation. Observation was done by one of the authors. Monomers that are classified constantly in the same position through time are more likely to have a specific orientation. An important consideration is distinguishing between singularity and multiply labeled classification, as it is not possible to determine orientation to singularity labeled. The variation of orientation for double labeled classification is determined through the sequential frames calculating the standard deviation of degree. Singularity labeled classification is not accounted for this purpose because of no directionality. Detections from different monomers through time are shown in Fig. 8. The average standard deviation for double label monomers with detected degree of orientations was 37.79 deg for the Gabor filter, 38.77 deg for the CNN model, and 29.9 deg for the eye observation.

In addition to noise and Poisson fluctuations in electron count, there may be other reasons for differences between successive images. The most likely is photobleaching as the sample is excited several times. Measuring the difference of the sequential images pixel by pixel over the eight times sample shows that the average signal decreases by only 2.18 counts with a standard deviation of 13.5 counts. Because the signal is so similar in every frame substantial photobleaching is unlikely. Imaged monomers were all adhered (completely or partially) to glass. It is possible that some of those monomers were only partially adhered (e.g., only one end of the monomer was adhered to the glass, whereas the other end was free). This would introduce limited variability in the monomer orientation. Therefore, the variability of monomers orientation over time shown in Fig. 8, can be due to detection method and/or partial adherence of monomers to glass.

## 4 Discussion and Conclusion

The use of epifluorescence microscopy allows for the detection and location of collagen monomers in solution. Multiple tags enable measurement of orientation. This investigation presents two algorithms to detect and analyze monomers smaller than the wavelength of light. With these measurements and algorithms, it is possible to observe collagen monomers dynamically in physiological conditions and in relation with collagen fibrils. The strong signal and comparatively low background provided by fluorescence allow for a nondestructive, high-contrast method to characterize these otherwise low-contrast objects. Using two fluorescent labels allows for the detection of monomer orientation. The machine-learning approach significantly outperforms the Gabor filters and appears to outperform human observers. Given the opacity of CNNs and the consequent lack of truthfulness and explainability, one may ask why the CNN behaves so well? We believe the reason lies in the large training set that teaches CNN to distinguish small departures from circular symmetry caused by Poisson fluctuations and noise from larger ones caused by the relative location of two fluorescent tags.

Collagen monomers in solution polymerize spontaneously into fibrils under physiological conditions,^20^ but the mechanisms that cause this to happen are not well understood. The use of a nondestructive method to measure the monomer orientation relative to nearby newly forming fibrils is a powerful tool to study the process of fibril formation in real time.

It is possible that collagen could cluster leading to accidental association of two labels on different by closely spaced monomers as a double-labeled one. However, we used an extremely small concentration of labeled monomers in our experiment to avoid monomers interaction. Also, labeled monomers that are out of focus could emit light that would be detected in an image, adding more noise than signal. In this work, we measured each monomer in multiple successive times. While we did not have the out of focus problem, it can be avoided by exchanging the solution after labeled monomers were absorbed to the surfaces (e.g., glass or fibril). In summary, this study provides evidence that the detection of collagen monomer orientation is possible using fluorescence microscopy with an application of a CNN. Such measurements could contribute to an increased understanding of monomer alignment during the process of collagen fibril formation. The next objective will inevitably be to determine orientation of labeled monomers on the surface of collagen fibrils in real time.

## References

[1] Brinckmann J. Bachinger H. P., Collagen: Primer in Structure, Processing A. Assembly, Springer, Berlin; New York (2005).

[2] Boedtker H. Doty P., “The native and denatured states of soluble collagen,” J. Am. Chem. Soc. 78(17), 4267–4280 (1956). JACSAT 0002-7863 10.1021/ja01598a024

[3] Van der Rest M. Garrone R., “Collagen family of proteins,” FASEB J. 5(13), 2814–2823 (1991). FAJOEC 0892-6638 10.1096/fasebj.5.13.1916105 1916105

[4] Parry D. A. Barnes G. R. Craig A. S., “A comparison of the size distribution of collagen fibrils in connective tissues as a function of age and a possible relation between fibril size distribution and mechanical properties,” Proc. R. Soc. London B Biol. Sci. 203, 305–321 (1978). 10.1098/rspb.1978.0107 33395

[5] Roveri N. et al., “X-ray diffraction study of bovine lens capsule collagen.” Biochim. Biophys. Acta 576(2), 404–408 (1979). BBACAQ 0006-3002 10.1016/0005-2795(79)90415-X 427197

[6] Mienaltowski M. J. Birk D. E., “Structure, physiology, and biochemistry of collagens,” Adv. Exp. Med. Biol. 802, 5–29 (2014). AEMBAP 0065-2598 10.1007/978-94-007-7893-1_2 24443018

[7] Magnusson S. P. Heinemeier K. M. Kjaer M., “Collagen homeostasis and metabolism,” in Metabolic Influences on Risk for Tendon Disorders, Ackermann P. Hart D., Eds., pp. 11–25, Springer, Cham (2016). 10.1007/978-3-319-33943-6_2 27535245

[8] Siadat S. M. et al., “Measuring collagen fibril diameter with differential interference contrast microscopy,” J. Struct. Biol. 213(1), 107697 (2021). JSBIEM 1047-8477 10.1016/j.jsb.2021.107697 33545351 PMC8754585

[9] Schmitt F. O. Hall C. E. Jakus M. A., “Electron microscope investigations of the structure of collagen,” J. Cell. Comp. Physiol. 20(1), 11–33 (1942). JCCPAY 0095-9898 10.1002/jcp.1030200103

[10] Gross J. Highberger J. H. Schmitt F. O., “Collagen structures considered as states of aggregation of a kinetic unit. The tropocollagen particle,” Proc. Natl. Acad. Sci. U. S. A. 40(8), 679–688 (1954). PNASA6 0027-8424 10.1073/pnas.40.8.679 16589538 PMC534141

[11] Siadat S. M., “On the mechanobiology of collagen growth and remodeling,” Dissertion, Northeastern University (2020).

[12] Siadat S. M. et al., “Development and validation of fluorescently labeled, functional type I collagen molecules,” bioRxiv 2021.03.26.437209 (2021). 10.1002/mabi.202100144 34856056

[13] Vrazhnov D. A. Nikolaev V. V. et al., “Analysis of collagen spatial structure using multiphoton microscopy and machine learning methods,” Biochem. Moscow 84, 108–123 (2019). 10.1134/S0006297919140074 31213198

[14] Paten J. A. et al., “Molecular Interactions between collagen and fibronectin: a reciprocal relationship that regulates de novo fibrillogenesis,” Chem 5, 2126–2145 (2019). 10.1016/j.chempr.2019.05.011

[15] Daugman J. G., “Uncertainty relation for resolution in space, spatial frequency, and orientation optimized by two-dimensional visual cortical filters,” J. Opt. Soc. Am. A 2(7), 1160–1169 (1985). JOAOD6 0740-3232 10.1364/JOSAA.2.001160 4020513

[16] Kamarainen J. K. Kyrki V. Kalviainen H., “Invariance properties of Gabor filter-based features-overview and applications,” IEEE Trans. Image Process. 15(5), 1088–1099 (2006). IIPRE4 1057-7149 10.1109/TIP.2005.864174 16671290

[17] Dabov K. et al., “Image denoising by sparse 3D transform-domain collaborative filtering,” IEEE Trans. Image Process. 16(8), 2080–2095 (2007). IIPRE4 1057-7149 10.1109/TIP.2007.901238 17688213

[18] Hu J. et al., “Squeeze-and-excitation networks,” in Proc. IEEE Conf. Comput. Vision and Pattern Recognit., pp. 7132–7141 (2018).

[19] Ronneberger O. Fischer P. Brox T., “U-net: convolutional networks for biomedical image segmentation,” Lect. Notes Comput. Sci. 9351, 234–241 (2015). LNCSD9 0302-9743 10.1007/978-3-319-24574-4_28

[20] Gross J. Kirk D., “The heat precipitation of collagen from neutral salt solutions: some rate-regulating factors,” J. Biol. Chem. 233(2), 355–360 (1958). JBCHA3 0021-9258 10.1016/S0021-9258(18)64764-7 13563501

